# Stereoselective (4 + 3) cycloadditions of allenyl ethers-furans by chiral auxiliaries inducing and evaluation of anti-breast cancer activity

**DOI:** 10.3389/fpls.2022.1087899

**Published:** 2022-12-21

**Authors:** Wenli Lei, Yuyang Song, Ai Long, Yanyan Que, Shuzhong He, Hang Zhong, Yang Chen

**Affiliations:** ^1^ School of Pharmaceutical Sciences, and Guizhou Engineering Laboratory for Synthetic Drugs, Guizhou University, Guiyang, Guizhou, China; ^2^ State Key Laboratory of Functions and Applications of Medicinal Plants, Guizhou Medical University, Guiyang, China; ^3^ State Key Laboratory Breeding Base of Green Pesticide and Agricultural Bioengineering, Ministry of Education, Guizhou University, Guiyang, China; ^4^ Key Laboratory of Green Pesticide and Agricultural Bioengineering, Ministry of Education, Guizhou University, Guiyang, China

**Keywords:** (4+3) cycloadditions, stereoselectivity, chiral auxiliaries, allenyl ethers, anti-breast cancer activity

## Abstract

The medicinal plants were wildly library of natural products in drug discovery. The most active molecules with seven-membered rings skeleton represent a challenge for construction. A stereoselectivity (4 + 3) cycloadditions between allenyl ethers and substituted furans induced by chiral auxiliaries has been investigated. And the results showed significant stereoselectivities and regioselectivities. The optical cycloadducts with an oxygen-substituted seven-membered ring framework were generated by removing chiral auxiliaries under acidic conditions. The antiproliferative activity of the novel compounds displayed moderate antiproliferative effects toward T47D cells.

## Introduction

**Figure 1:**
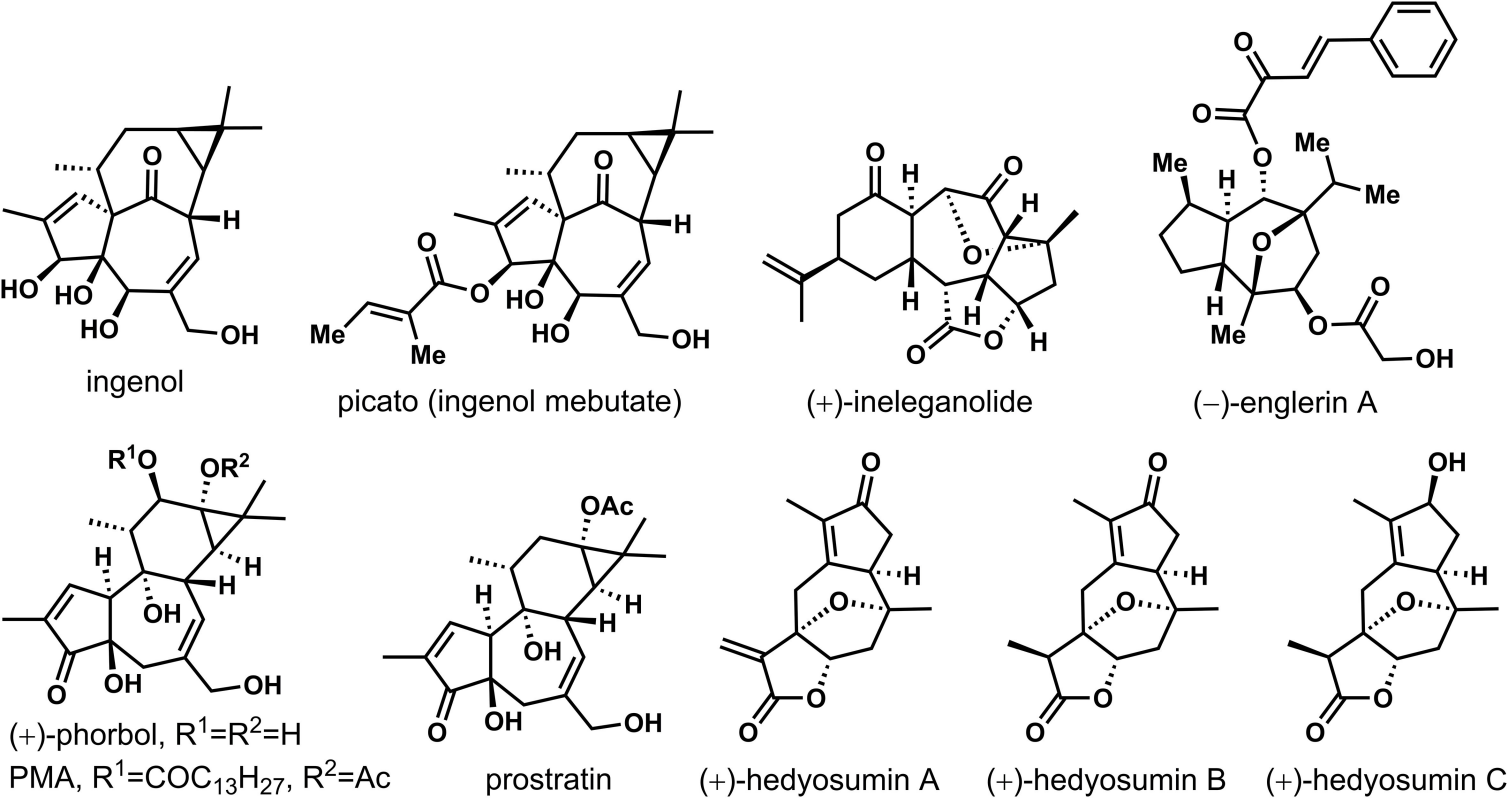
Several active natural products with seven-membered carbon rings.

Natural products are important sources of drug discovery. The bioactive chemical constituents in medicinal plants are the effective material bases for the disease prevention and treatment (Harvey, 2008; Newman and Cragg, 2020; Atanasov et al., 2021). However, due to the low natural content of many natural products, separation and extraction difficulties and other factors that limit their subsequent activity research. How to achieve efficient synthesis of trace active natural products has always been an important scientific problem to be solved (Kim et al., 2021; Li et al., 2022). In recent years, as scientists pay more and more attention to the field of total synthesis of natural products, the total synthesis of many natural products has been realized (Nicolaou et al., 2019). The seven-membered rings construction represents a more challenging task than the preparation of the smaller five- or six-membered analogues due to their higher ring strain and to entropy issues (Foley and Maguire, 2010; Nguyen et al., 2013). In particular, this motif is ubiquitous in natural products with significant biological activities. As shown in **Figure 1**, (+)-ingenol was isolated from *Euphorbia ingens* for the first time, then from *Euphorbia peplus* (Evans and Kinghorn, 1977; Foley and Maguire, 2010). Its derivatives showed significant anti-cancer and anti-HIV activities, such as , picato (ingenol mebutate), which was recently approved by the FDA as a first-in-class drug for the topical treatment of actinic keratosis, and a precancerous skin lesions (Lebwohl et al., 2012). Highly oxygenated (+)-ineleganolide, which isolated from the soft coral *Sinularia inelegans* in 1999, showed anti-leukemic (Duh et al., 1999). Phobosol, which was first isolated from seeds of *Croton tiglium* in 1934, was one of the most famous natural products of Tigrian family (Flaschenträger and v. Wolffersdorff, 1934). Esters of phorbol showed tumorigenic when protein kinase C was activated. Phorbol-12-myristate-13-acetate (PMA) has been most used as a biomedical research tool in carcinogenic models (Wender et al., 1989; Wender and McDonald, 1990; Wender et al., 1997; Kawamura et al., 2016). Prostratin was also one of the natural products of the Tigliane family that had potent anti-HIV activity, even could fight against latent viral hosts. It was found in the bark of mamala tree *Homalanthus Nutans* of the Euphorbiaceae family, and traditionally used by the Samoan natives to treat hepatitis. Biological evaluation of sesquiterpenoid natural product (−)-englerins A, isolated from the Tanzanian plant *Phyllanthus Engleri* demonstrated strong and selective nanomolar cytotoxicity against various renal cancer cell lines. Nevertheless, some natural products cannot systematically carry out pharmacological activity research because of the lack of natural resources. Guaiacolide (+)-hedyotin A-C isolated from *Hedyotis Orientalis* had limited efforts to reveal its complete biological characteristics due to its deficient resource availability (Wang et al., 2015).

As one of the most direct methods for the construction of seven-membered carbocycles, the (4+3) cycloaddition between oxyallyl cations and dienes had attracted extensive attentions in the scientific community (Montana and Grima, 2001; Lohse and Hsung, 2011; Okamoto et al., 2018; Yin et al., 2018). Specifically, significant advances have been made by employing heteroatoms such as halogens, (Harmata et al., 1991; Lee and Cha, 1999) oxygen, (Masuya et al., 1998; Lee and Cha, 1999; Hayakawa and Shimizu, 2000; Saez et al., 2003; Sáez et al., 2005) sulfur, (Harmata et al., 1991; Hardinger et al., 1995; Fuchigami et al., 2012) and nitrogen (Myers and Barbay, 2001; Wei et al., 2003; He et al., 2014) to modify the stability and reactivity of oxyallyl species as well as to introduce an electronic bias in these intermediates that can lead to high regioselectivity and stereoselectivity.

**Scheme 1:**
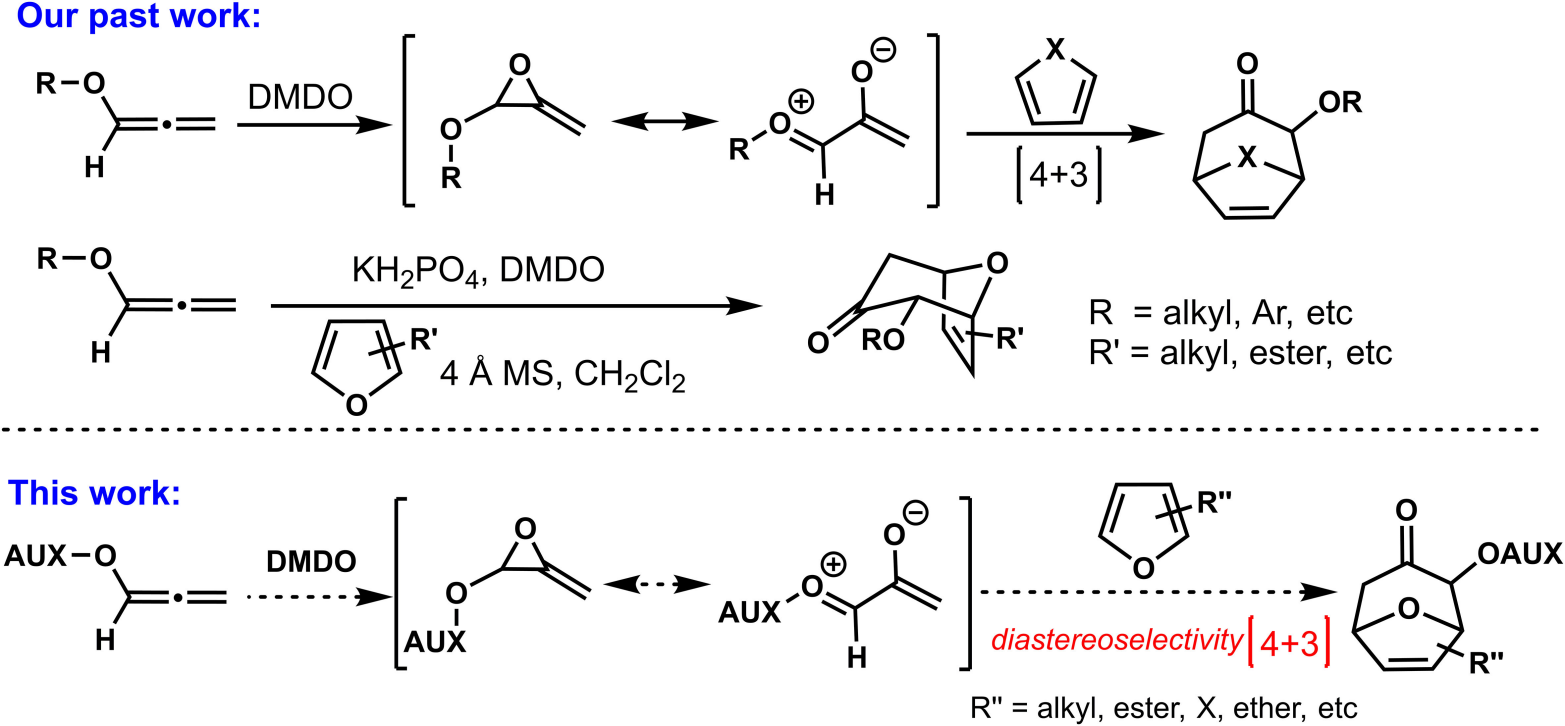
Strategies for (4+3) cycloaddition.

As shown in **Scheme 1**, in 2019, our group developed a series of (4 + 3) cycloadditions based on oxygen-stabilized oxyallyl intermediates derived from epoxidations/ring-opening of allenyl ethers, opening the curtain on the involvement of oxyallyl cations in (4 + 3) cycloadditions (Huang et al., 2020). Although the (4 + 3) cycloadditions based on allenyl ethers had been successfully established, we believed that the stereoselective control of allenyl ethers (4 + 3) cycloadditions was necessary and meaningful, which could get a new practical method for the synthesis of optical seven-member carbon rings with oxygen atom substitution. As a continuation of allenyl ethers (4 + 3) cycloadditions, we reported the present progress of stereoselective (4 + 3) cycloadditions by assembling chiral auxiliaries on allene, and the cytotoxicities of the cycloadducts *in vitro* against a sort of human cancer cell lines were evaluated by MTT assay.

## Results and discussion

### (4 + 3) Cycloaddition: Screening of chiral auxiliaries

Stereoselective synthesis induced by chiral auxiliaries had been well developed (Whitesell et al., 1985; Potin et al., 1990; Harrington and Tius, 2000; Harrington et al., 2002). As shown in **Table 1**, six chiral auxiliaries were introduced on allene to explore the optimal stereoselectivity of (4 + 3) cycloadditions with furan as dienes. Using the classic allenyl ethers (4 + 3) reaction condition (KH_2_PO_4_, DMDO, CH_2_Cl_2_, −30°C) (Huang et al., 2020), the reaction did not give the desired products, but led to a mixture of unidentifiable side-products (entries 1 ~ 2) when induced with **Aux-1** and **Aux-2**. When induced with **Aux-3** and **Aux-4**, (4 + 3) products were obtained with *d.r.* (50: 50) and 54% yield respectively. In order to improve the stereoselectivity, the situation of **Aux-5** induction was further explored. Encouragingly, the cycloadduct was obtained with 60% yield and the *d.r.* value increased to 84:16 when **Aux-5** was induced (entry 5).

**Table 1:** Screening for chiral auxiliary.

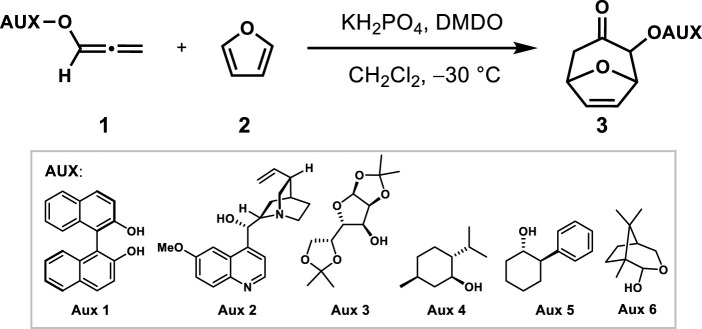
Entry	AUX	Compound 3	
		yield (%)	*d.r.*
1	**Aux-1**	0	0
2	**Aux-2**	0	0
3	**Aux-3**	50	50:50
4	**Aux-4**	54	50:50
5	**Aux-5**	60	84:16
6	**Aux-6**	30	100:00

Considering the removal of the chiral auxiliary in the later stage and further improving the stereoselectivity, the camphor-derived chiral auxiliary **Aux-5** with glycosidic bonds was selected, which was developed to asymmetric Nazarov cyclization by Tius in 2007 (Harrington et al., 2002). Under the induction of the chiral auxiliary, the cycloadduct **3a** could be successfully obtained with a yield of 30% and a diastereoselectivity ratio of 100: 0 (entry 6), and an uncyclized allene reduction products was also produced, which led to low yields of cycloaddition products. The possible reason was the high activation energy caused by temperature.

### (4+3) Cycloaddition: Expansion of furans

**Scheme 2:**
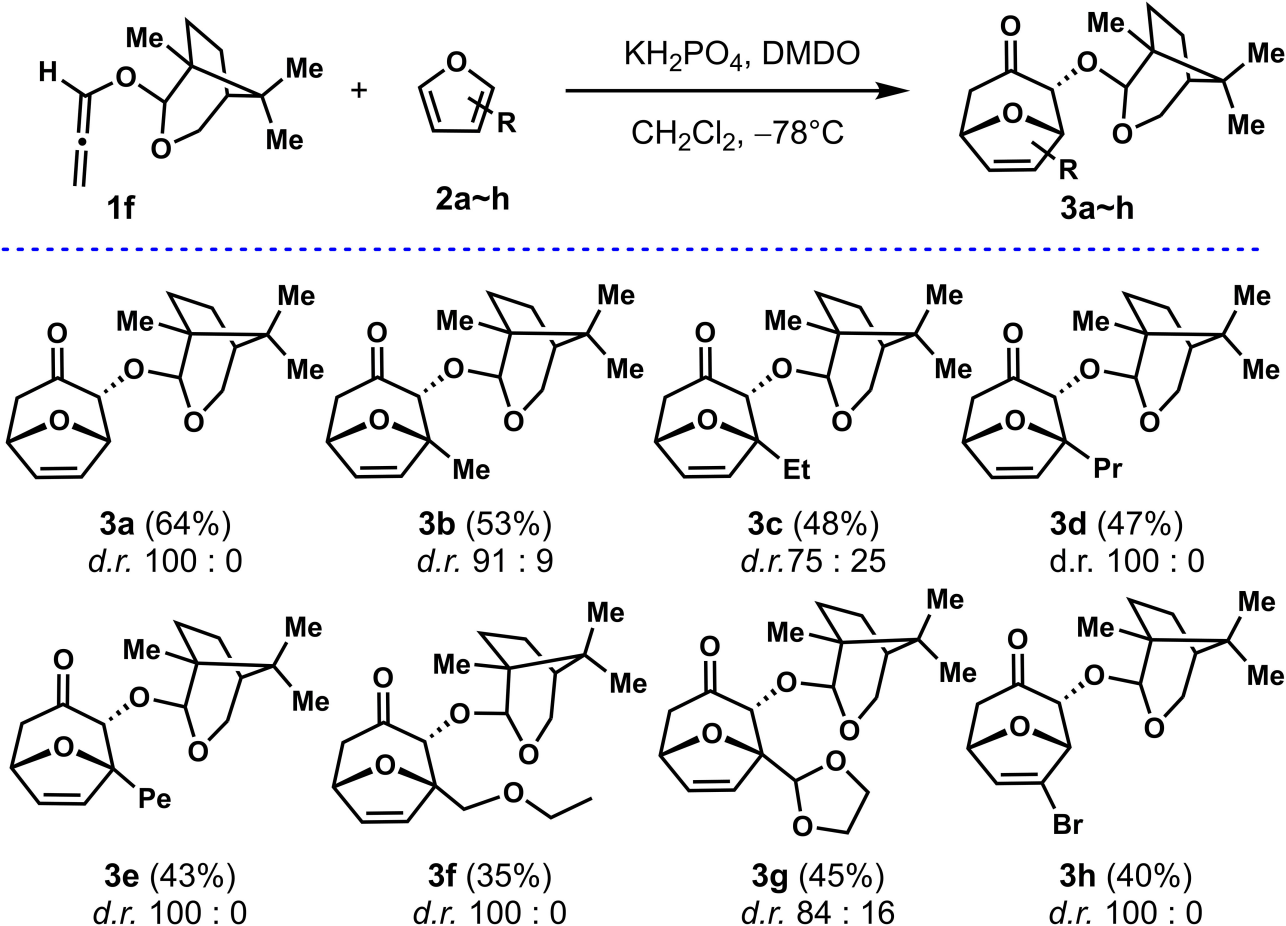
Cycloaddition of 1f with substituted furans.

With a highly stereoselective auxiliary **Aux-6** in hand, allenyl ethers **1f** was used as a common substrate for the (4 + 3) cycloadditions of furans with different substituents to explore the adaptability of its substrate. As shown in **Scheme 2**, firstly, to improve the reaction efficiency of the (4 + 3) cycloadditions, the temperature was reduced to −78°C with other conditions unchanged, the reaction yield was almost doubled, for instance, **3a** were obtained with *d.r.* (100: 0) and 64% yield respectively. With the optimized conditions in hand, the scopes of regioselectivity and stereoselectivity of allenyl ethers (4 + 3) cycloaddition induced by **Aux-6** was explored. Different substituted furans were employed for reaction with **1f**. When 2-substituted furans were explored for the (4 + 3) cycloaddition, the carbon chain increased to five carbons, the yield remained between 43% to 53% (entries 2~4). Cycloaddition of 2-methylfuran and 2-ethylfuran with **1f** provided cycloadducts **3b** and **3c** with moderate diastereoselectivity, specifically decreased to 91: 19 and 75: 25, while other alkyl substitutions, remained unchanged. Using methyl 2-furoate and 2-furonitrile as the substrate led to a mixture of unidentifiable isomers and side-product. The cycloadducts can be obtained with moderate yield and the diastereoselectivity was not affected when the second position of furan was substituted by alkyl ether (entries 7~8).

To further study the substrate scope and stereoselectivity of the (4 + 3) cycloadditions, we also employed 3-substituted for the (4 + 3) cycloadditions. The cycloadduct was not obtained by using methyl 3-furoate as the diene, while 3-bromofuran was explored as the substrate, the cycloadduct **3h** was obtained at 40% yield with the diastereoselectivity of 100: 0. The absolute configuration of the cycloadducts obtained was established by X-ray analysis of enantiopure samples of compound 3h. The configuration of all other adducts **3a~3g**, was established by assuming the same stereochemical outcome for all reactions based on mechanistic analogy.

### Chiral auxiliary cleavage of cycloadduct

**Scheme 3:**
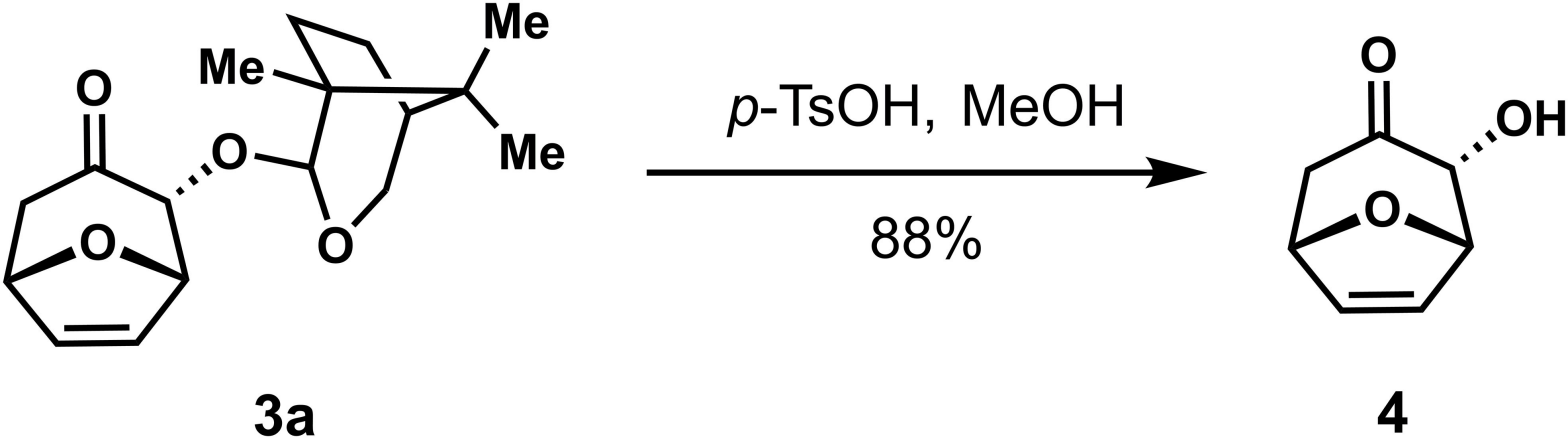
Removal of the chiral auxiliary on the cycloadduct.

To demonstrate the synthetic potential of this stereoselective (4 + 3) reaction, the possibility of removing the chiral auxiliary on the cycloadducts was explored. The chiral auxiliary was easily removed from the cycloadducts under acidic conditions to provide α-hydroxyl cycloheptanone with high stereoselectivity (**Scheme 3**).

### Evaluation of anti-breast cancer activity of cycloadducts

Since 2020, breast cancer has become the highest morbidity malignancy in the world, (Sung et al., 2021), an estrogen-dependent (estrogen receptor positive, ER^+^) human breast cancer cell line T47D was selected to evaluate the antiproliferative activities of these novel cycloadducts with Ribociclib (an approval CDK4/6 inhibitor for advanced ER^+^ breast cancer treatment) as a positive control. As shown in **Table 2**, **3a, 3b, 3g, 3h** and **4** displayed moderate antiproliferative effects towards T47D cells with inhibitory rates higher than or equal to 50% at 100 μmol/L, while **3c, 3d, 3e** and **3f** featuring a *C*2-electron-donor substituent were less potent than their counterparts. This indicated that a long chain electron-donating substituent at *C*2-position was detrimental to the antiproliferative effects of the novel compounds. In contrary, an electron-withdrawing substituent at *C*2 (8) or *C*3 (6) position exhibited more potent inhibitory activities in T47D cells. Intriguingly, compound **4** had the simplest scaffold and its maintenance of antiproliferative activity was observed, showing great potential for further modification.

**Table 2:** The inhibitory rates of the target compounds on breast cancer cells (100 μmol/L).

Cycloadducts	Inhibitory rates (%)
	T47D
**3a**	52.17
**3b**	50.32
**3c**	38.69
**3d**	31.96
**3e**	48.61
**3f**	46.48
**3g**	61.32
**3h**	52.31
**4**	52.97

Ribociclib was used as positive control.

## Materials and methods

### Equipment and materials

All reactions were performed in flame-dried glassware under nitrogen atmosphere. Solvents were distilled prior to use. Reagents were used as purchased from Aladdin, Macklin, Innochem, or TLC unless otherwise noted. Chromatographic separations were performed using Silica Gel, AR, 200-300 mesh. ^1^H and ^13^C NMR spectra were obtained on a Bruker (Avance) 400 MHz NMR instrument using CDCl_3_ as the solvent, which was provided by School of Pharmaceutical Sciences, Guizhou University. Infrared spectra were obtained on a Shimadzu FT-IR-8400S spectrometer as KBr pellets. Optical rotations were obtained on a Insmark digital polarimeter using the sodium (589 nm, D line) lamp and are reported as follows: (c = g/100 mL, solvent). TLC analysis was visualized using UV, *p*-anisoladehyde and phosphomolybdic acid stains. High-resolution mass spectra were obtained using AB SCIEX X500R QTOF. All spectral data obtained for new compounds are reported here.

Biological reagents such as 3-(4,5-Dimethylthiazol-2-yl)-2,5- diphenyltetrazolium bromide (MTT), penicillin and streptomycin were obtained from Sigma. In addition, fetal bovine serum (FBS), phosphate buffered saline (PBS), 1640 medium, DMEM and trypsin were purchased from Biosera and Adriamycin was obtained from EBEWE Pharma. The antiproliferative activity results were recorded on a Bio-Rad microplate reader (Model 680).

### Synthetic procedures for chiral auxiliary AUX-6 and allenyl ether 1f

Compound **AUX-6** was prepared to correspond to literature, (Harrington et al., 2002) and the NMR data was corresponding to ones described in the literature.

**Scheme 4:**
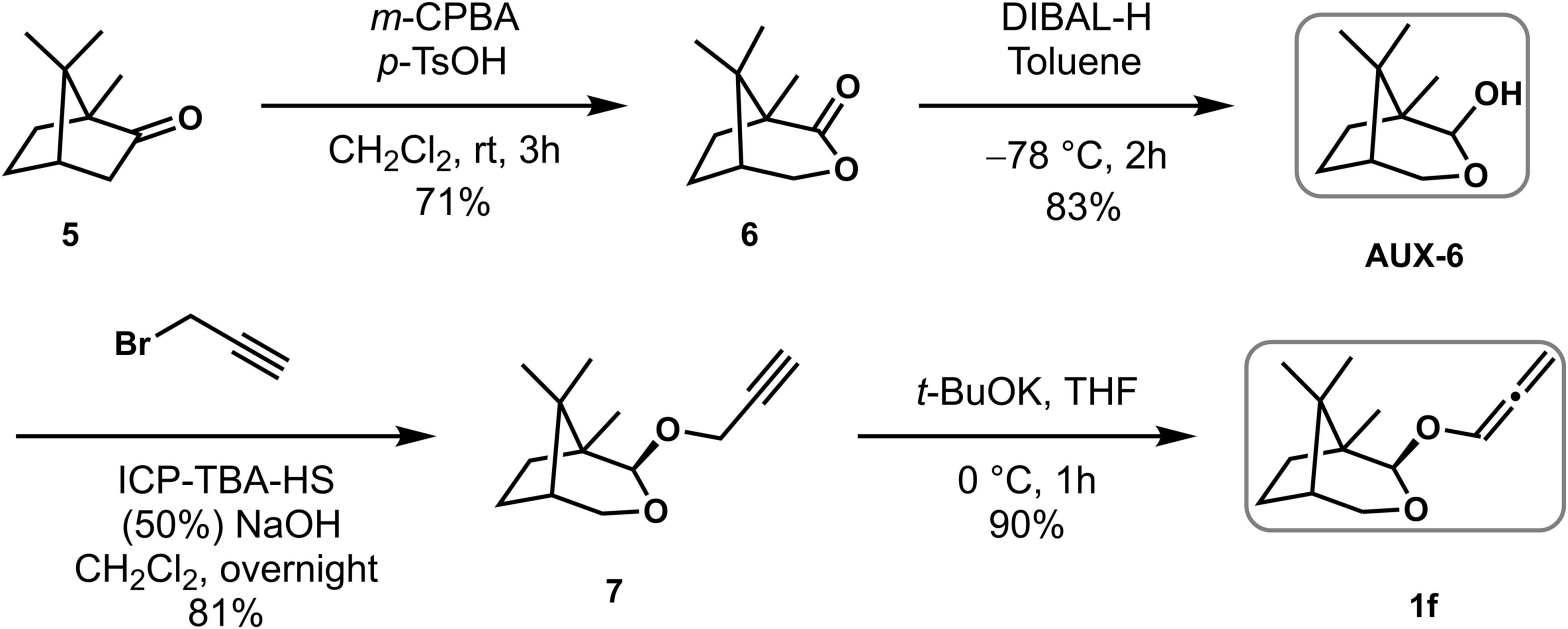
Preparation of chiral auxiliary Aux-6 and allenyl ether 1f.

As shown in **Scheme 4,** a pH 7 saturated phosphate buffer was made from H_2_O (70 mL), NaH_2_PO_4_ (3.8 g) and Na_2_HPO_4_ (5.6 g). Na_3_PO_4_ was added to a portion of the phosphate buffer together with some H_2_O until pH 8. *m-CPBA* was dissolved in diethyl ether and this organic phase was stirred for 1 h with a generous quantity of the modified phosphate buffer. The organic phase was separated, washed with brine (1×), dried over MgSO_4_ and evaporated in small portions at room temperature under reduced pressure. Purified and dried *m*-CPBA (4.3 g, 24.8 mmol) was suspended / partially dissolved in dichloromethane (19.8 mL) and trifluoromethanesulfonic acid (0.9 mL, 9.9 mmol) was added slowly, finally making a clear and slightly yellow solution. L-Camphor **5** (1.5 g, 9.9 mmol) was added in one portion and the solution left stirring for 3 h at room temperature. The resultant thick reaction mixture was quenched by the slow addition of aqueous NaOH (3 M) until pH 12. The aqueous phase was separated, extracted with EtOAc (3×) and the combined organic extracts were washed with saturated aqueous NaHCO_3_ (2×), brine (2×), dried over MgSO_4_. After filtration and concentration, the crude product was purified using silica gel flash column chromatography [eluent: 3.3% EtOAc/Petroleum ether] to give white crystalline lactone **6** (1.2 g, 71% yield). ^1^H NMR (400 MHz, CDCl_3_) *δ* 4.57 – 4.39 (m, 1H), 4.11 (dd, *J* = 10.8, 1.2 Hz, 1H), 2.20 – 2.04 (m, 2H), 1.97 – 1.66 (m, 3H), 1.18 (s, 3H), 1.10 (s, 3H), 0.98 (s, 3H).

To a solution of lactone **6** (1.2 g, 7.1mmol) in toluene at –78 °C was added DIBAL-H (9.5 mL, 14.2 mmol) *via* syringe pump for 2 h. After the addition, the reaction mixture was stirred for 1h, quenched with acetone (3.6 mL), warmed to room temperature, diluted with aqueous potassium sodium tartrate (4.5 g, 21.3 mmol in 22.5 mL H_2_O), stirred for 1 h and then diluted with EtOAc, sat. aq. NaCl and water. The aqueous phase was extracted with EtOAc (3×) and the combined organic extracts were washed with brine (2×), dried over MgSO_4_. After filtration and concentration, the crude product was purified using silica gel flash column chromatography [eluent: 4% EtOAc/Petroleum ether] to give white and crystalline lactol **Aux-6** (1.0 g, 83% yield). ^1^H NMR (400 MHz, CDCl_3_) *δ* 4.97 – 4.85 (m, 1H), 4.07 (dt, *J* = 10.9, 1.3 Hz, 1H), 3.52 (dd, *J* = 10.9, 2.5 Hz, 1H), 3.12 – 2.97 (m, 1H), 2.03 – 1.77 (m, 2H), 1.70 – 1.48 (m, 3H), 1.08 (s, 3H), 0.91 (s, 3H), 0.87 (s, 3H).

Crystalline lactol **Aux-6** (1.0 g, 5.9 mmol) and tetra-*n*-butylammonium hydrogen sulfate (0.30 g, 5.2 mmol) were dissolved in CH_2_Cl_2_ (23 mL) and cooled to 0°C. Aqueous sodium hydroxide (50%, 23 mL) was cooled to 0°C and added to the solution of the lactol and phase transfer catalyst. The mixture was stirred for 5 min and propargyl bromide (5.0 mL, 5.9 mmol) was added. The thick reaction mixture was stirred vigorously for 6 h at rt and diluted with EtOAc and water. The aqueous phase was extracted with EtOAc (3×), the combined organic extracts were washed with brine (2×), dried over MgSO_4_ and concentrated under vacuum to give a dark brown oil, which after purification by silica gel flash column chromatography [eluent: 0.5% EtOAc/Petroleum ether] gave a yellow oil of alkyne **7** (1.0 g, 81% yield).^1^H NMR (400 MHz, CDCl_3_) *δ* 4.74 (d, *J* = 0.9 Hz, 1H), 4.33 (dd, *J* = 12.2, 2.4 Hz, 2H), 4.04 (d, *J* = 10.9 Hz, 1H), 3.53 (dd, *J* = 10.9, 2.5 Hz, 1H), 2.40 (d, *J* = 2.4 Hz, 1H), 2.07 (ddd, *J* = 13.2, 9.9, 4.6 Hz, 1H), 1.94 – 1.73 (m, 1H), 1.69 – 1.45 (m, 2H), 1.41 – 1.20 (m, 1H), 1.09 (s, 3H), 0.90 (s, 3H), 0.86 (s, 3H).

Compound 1f was prepared to correspond to literature, (Huang et al., 2020) and the NMR data was corresponding to ones described in the literature.

To a solution of alkyne **7** (1.00 g, 4.8 mmol) in THF (48 mL) was added t-BuOK (1.0 M solution in THF, 7.2 mL, 7.2 mmol) at 0°C. The reaction was stirred at rt for 1 h before being concentrated under reduced pressure. Subsequently, the residue was first suspended in CH_2_Cl_2_ and then filtered through Celite^TM^. The filtrate was concentrated under reduced pressure and the crude residue was purified using silica gel flash column chromatography [eluent: 0.5% EtOAc/Petroleum ether] to give a yellow oil allenyl ether **1f** (0.9 g, 90% yield).^1^H NMR (400 MHz, CDCl_3_) *δ* 6.70 (t, *J* = 6.0 Hz, 1H), 5.42 (dd, *J* = 12.9, 6.1 Hz, 2H), 4.82 (s, 1H), 4.06 (d, *J* = 10.9 Hz, 1H), 3.59 (dd, *J* = 10.9, 2.4 Hz, 1H), 2.17 (s, 1H), 1.96 – 1.78 (m, 1H), 1.69 – 1.52 (m, 3H), 1.19 – 0.76 (m, 9H).

### Synthetic procedures for (4 + 3) cycloaddition of allenyl ethers-furans

To a solution of allenyl ether **1f** (1.0 equiv) in CH_2_Cl_2_ (0.05 M) containing anhydrous 4Å MS were added KH_2_PO_4_ (5.0 equiv) and furan **2a~h** (10.0 equiv) at –78°C. After which, a dry-ice chilled solution of DMDO (5.0 equiv, 0.08 M solution in CH_2_Cl_2_,) was added *via* syringe pump over 1.5 h. The reaction mixture was stirred at this temperature for another 1 h before being filtered through Celite^TM^. The filtrate was concentrated under reduced pressure and the crude residue was purified using silica gel flash column chromatography [eluent: EtOAc/Petroleum ether] to give a desired yellow oil cycloadduct **3a~h**.

**3a**: 26 mg (64%); yellow oil; ^1^H NMR (400 MHz, CDCl_3_) *δ* 6.31 (ddd, *J* = 26.9, 6.1, 1.7 Hz, 2H), 5.08 (dd, *J* = 5.2, 1.8 Hz, 1H), 5.00 (d, *J* = 5.0 Hz, 1H), 4.81 (s, 1H), 4.39 (d, *J* = 5.2 Hz, 1H), 3.99 (d, *J* = 10.9 Hz, 1H), 3.50 (dd, *J* = 10.9, 2.5 Hz, 1H), 2.75 (dd, *J* = 15.5, 4.9 Hz, 1H), 2.34 (d, *J* = 15.5 Hz, 1H), 2.06 (td, *J* = 9.8, 5.0 Hz, 1H), 1.91 – 1.76 (m,1H), 1.67 – 1.45 (m, 3H), 1.06 (s, 3H), 0.94 (s, 3H), 0.89 (s, 3H); ^13^C NMR (100 MHz, CDCl_3_) *δ* 204.5, 134.2, 132.4, 103.2, 82.5, 80.7, 78.2, 68.4, 47.4, 45.8, 45.4, 42.5, 28.5, 26.0, 24.7, 17.7, 13.6; IR (KBr) cm^-1^ 2962m, 2924m, 2869m, 1725m, 1463w, 1361w, 1322w, 1258w, 1175w, 1095s, 1012s, 964s, 804m, 723s; HRMS (ESI^+^): C_17_H_24_O_4_ for [M+Na]^+^, calculated 315.1572, found 315.1566; [α]_D_^25^ = + 9.921 (c 1.00, CHCl_3_).

**3b**: 15 mg (52% yield); yellow oil; ^1^H NMR (400 MHz, CDCl_3_) *δ* 6.26 (dd, *J* = 5.9, 1.8 Hz, 1H), 6.07 (d, *J* = 5.9 Hz, 1H), 5.10 – 5.00 (m, 1H), 4.81 (s, 1H), 4.35 (d, *J* = 5.2 Hz, 1H), 3.98 (d, *J* = 10.9 Hz, 1H), 3.49 (dd, *J* = 10.9, 2.5 Hz, 1H), 2.55 (s, 1H), 2.39 (d, *J* = 15.3 Hz, 1H), 2.10 – 1.99 (m, 1H), 1.91 – 1.77 (m, 1H), 1.63 – 1.52 (m, 3H), 1.49 (s, 3H), 1.05 (s, 3H), 0.93 (s, 3H), 0.88 (s, 3H); ^13^C NMR (100 MHz, CDCl_3_) *δ* 204.97, 137.38, 132.21, 103.07, 84.80, 81.21, 80.96, 68.40, 51.73, 47.42, 45.44, 42.54, 28.46, 25.95, 24.75, 22.77, 17.68, 13.62; IR (KBr) cm^-1^ 2962m, 2927m, 2866m,1725m, 1460w, 1383w, 1325w, 1178w, 1115s, 1076s, 1006s, 830w, 736m, 727m; HRMS (ESI^+^): C_18_H_26_O_4_ for [ M+Na]^+^, calculated 329.3918, found 329.17248; ${\left[\text{α}\right]}_{D}^{25}$ = + 17.105, (c 0. 67, CHCl_3_)

**3c**: 21 mg (48% yield); yellow oil; ^1^H NMR (400 MHz, CDCl_3_) *δ* 6.27 (d, J = 6.0 Hz, 1H), 6.07 (d, J = 5.8 Hz, 1H), 5.07 (d, J = 5.2 Hz, 1H), 4.82 (s, 1H), 4.37 (d, J = 5.2 Hz, 1H), 3.98 (d, J = 11.0 Hz, 1H), 3.49 (d, J = 11.0 Hz, 1H), 2.55 (d, J = 15.2 Hz, 1H), 2.36 (d, J = 15.3 Hz, 1H), 2.07 (ddt, J = 14.8, 9.4, 5.3 Hz, 2H), 1.87 – 1.76 (m, 2H), 1.65 – 1.46 (m, 3H), 1.06 (s, 3H), 0.98 (t, J = 7.5 Hz, 3H), 0.94 (s, 3H), 0.89 (s, 3H); ^13^C NMR (100 MHz, CDCl_3_) *δ* 205.2, 136.0, 132.5, 103.1, 88.1, 81.6, 80.9, 68.4, 50.0, 47.4, 45.4, 42.5, 28.9, 28.5, 26.0, 24.8, 17.7, 13.6, 8.8; IR (KBr) cm^-1^ 2959m, 2924m, 2850m, 1726m, 1470m, 1169w, 1120s, 1069s, 1008s, 967w, 826w, 804w, 733s; HRMS (ESI^+^): C_19_H_28_O_4_ for [ M+Na]^+^, calculated 343.4188, found 343.18769; [α]^D25^= + 16.221, (c 1.00, CHCl_3_).

**3d**: 22 mg (47% yield); yellow oil; ^1^H NMR (400 MHz, CDCl_3_) *δ* 6.26 (dd, *J* = 5.9, 1.8 Hz, 1H), 6.08 (d, *J* = 6.0 Hz, 1H), 5.06 (dd, *J* = 5.2, 1.9 Hz, 1H), 4.82 (s, 1H), 4.37 (d, *J* = 5.2 Hz, 1H), 3.99 (d, *J* = 10.9 Hz, 1H), 3.50 (dd, *J* = 10.9, 2.5 Hz, 1H), 2.54 (s, 1H), 2.38 (s, 1H), 2.11 – 2.03 (m, 2H), 1.87 – 1.80 (m, 1H), 1.77 – 1.70 (m, 2H), 1.62 – 1.55 (m, 2H), 1.51 (m, 2H), 1.06 (s, 3H), 0.97 (s, 3H), 0.94 (m, 3H), 0.90 (d, *J* = 2.4 Hz, 3H). ^13^C NMR (100 MHz, CDCl_3_) *δ* 205.3, 136.3, 132.2, 103.1, 87.7, 81.6, 80.8, 68.4, 50.3, 47.4, 45.4, 42.5, 38.3, 28.5, 26.0, 24.8, 17.7, 17.4, 14.4, 13.6; IR (KBr) cm^-1^ 2961w, 2930s, 2852w, 1730m, 1604w, 1513w, 1467m, 1393m, 1263m, 1183w, 1085s, 1008s, 962m, 825m, 795m, 764m, 706m, 661m, 559m; HRMS (ESI^+^): C_20_H_30_O_4_ for [ M+Na]^+^, calculated 357.4458, found 357.20370; [α]_D_^20^= + 140.898, (c 0.20, CHCl_3_).

**3e**: 22 mg (43% yield); yellow oil; ^1^H NMR (400 MHz, CDCl_3_) *δ* 6.27 (dd, *J* = 6.0, 1.8 Hz, 1H), 6.08 (d, *J* = 6.0 Hz, 1H), 5.12 – 5.03 (m, 1H), 4.82 (s, 1H), 4.37 (d, *J* = 5.2 Hz, 1H), 3.99 (dd, *J* = 11.0, 1.3 Hz, 1H), 3.50 (dd, *J* = 10.9, 2.5 Hz, 1H), 2.56 (d, *J* = 15.2 Hz, 1H), 2.44 – 2.34 (m, 1H), 2.08 (ddd, *J* = 12.9, 9.8, 4.7 Hz, 2H), 1.90 – 1.78 (m, 1H), 1.74 – 1.48 (m, 3H), 1.06 (s, 3H), 0.95 (s, 4H), 0.93 – 0.85 (m, 8H); ^13^C NMR (100 MHz, CDCl_3_) *δ* 205.2, 136.4, 132.3, 103.1, 87.7, 81.6, 80.8, 68.4, 50.4, 47.4, 45.5, 42.5, 36.1, 32.1, 28.5, 26.0, 24.8, 23.7, 22.6, 17.7, 14.1, 13.6; IR (KBr) cm^-1^ 2957m, 2924m, 2877m, 1731m, 1492m, 1457m, 1396w, 1362w, 1309w,1274m, 1206w, 1184m, 1122m, 1082s, 972s, 893w, 817w, 738w, 708w, 601w; HRMS (ESI^+^):C_22_H_34_O_4_ for [ M+Na]^+^, calculated 385.4998 found 385.47654; [α]_D_^25^ = + 121.312, (c 0.10, CHCl_3_).

**3f**: 17 mg (35% yield); yellow oil; ^1^H NMR (400 MHz, CDCl_3_) *δ* 6.34 (dd, *J* = 6.0, 1.8 Hz, 1H), 6.14 (d, *J* = 6.0 Hz, 1H), 5.12 (d, *J* = 5.3 Hz, 1H), 4.82 (s, 1H), 4.39 (d, *J* = 5.2 Hz, 1H), 3.99 (d, *J* = 10.9 Hz, 1H), 3.66 (s, 2H), 3.61 (dd, *J* = 7.0, 4.4 Hz, 1H), 3.51 (d, *J* = 2.5 Hz, 1H), 2.77 (d, *J* = 15.5 Hz, 1H), 2.38 (d, *J* = 15.4 Hz, 1H), 2.07 (ddd, *J* = 13.9, 9.7, 4.7 Hz, 1H), 1.84 (d, *J* = 6.0 Hz, 1H), 1.65 – 1.49 (m, 3H), 1.06 (s, 3H), 0.94 (s, 3H), 0.92 – 0.85 (m, 9H); ^13^C NMR (100 MHz, CDCl_3_) *δ* 204.9, 134.7, 133.1, 103.2, 87.3, 81.4, 81.2, 72.3, 68.4, 67.4, 47.4, 47.3, 45.4, 42.5, 28.5, 25.9, 24.8, 17.7, 15.0, 13.6; IR (KBr) cm^-1^ 2963m, 2959m, 2857m, 1724m, 1467m, 1387w, 1263m, 1078s, 1011m, 965w,829m, 801m, 794m; HRMS (ESI^+^): C_20_H_30_O_5_ for [M+Na]^+^, calculated 373.4448, found 373.19847; [α]_D_^25^ = + 3.831, (c 0.10, CHCl_3_).

**3g**: 23 mg (45% yield); yellow oil; ^1^H NMR (400 MHz, CDCl_3_) *δ* 6.38 (d, *J* = 1.8 Hz, 1H), 6.20 (d, *J* = 6.0 Hz, 2H), 5.16 (dd, *J* = 5.2, 1.8 Hz, 2H), 5.05 (s, 2H), 4.82 (s, 2H), 4.39 (d, *J* = 5.2 Hz, 2H), 4.10 – 3.94 (m, 6H), 3.49 (dd, *J* = 11.0, 2.5 Hz, 2H), 2.76 (d, *J* = 15.5 Hz, 2H), 2.42 (d, *J* = 15.5 Hz, 2H), 2.12 – 2.00 (m, 3H), 1.83 (dt, *J* = 11.8, 5.8 Hz, 2H), 1.63 – 1.50 (m, 5H), 1.06 (s, 6H), 0.94 (s, 7H), 0.90 (d, *J* = 2.5 Hz, 11H); ^13^C NMR (100 MHz, CDCl_3_) *δ* 204.4, 133.7, 133.4, 103.2, 103.1, 88.2, 81.5, 81.3, 68.4, 65.9, 65.63, 5.40, 45.4, 44.8, 42.5, 28.5, 25.9, 24.7, 17.69, 13.6; IR (KBr) cm^-1^ 2953m, 2918m, 2871m, 2853m, 1726m, 1467w, 1373w, 1315w, 1257w, 1176w, 1109s, 1078s, 1014m, 964m, 938m, 838w, 798w, 740m, 613w, 491w; HRMS (ESI^+^): C_20_H_28_O_6_ for [ M+Na]^+^, calculated 387.4278, found 387.17796; ${[\text{α]}}_{\text{D}}^{20}$ = + 73.604, (c 0.12, CHCl_3_).

**3h**: 21 mg (40% yield); white solid; mp 80.7~81.2°C; ^1^H NMR (400 MHz, CDCl_3_) *δ* 6.38 (d, *J* = 2.0 Hz, 1H), 4.97 (d, *J* = 5.1 Hz, 1H), 4.95 – 4.93 (m, 1H), 4.84 (s, 1H), 4.52 (d, J = 5.1 Hz, 1H), 4.00 (d, *J* = 10.9 Hz, 1H), 3.52 (dd, *J* = 10.9, 2.5 Hz, 1H), 2.77 (d, *J* = 4.8 Hz, 1H), 2.43 (d, *J* = 15.7 Hz, 1H), 2.24 (ddd, *J* = 13.8, 9.9, 4.6 Hz, 1H), 1.97 – 1.78 (m, 1H), 1.69 – 1.42 (m, 3H), 1.06 (s, 3H), 0.97 (s, 3H), 0.90 (s, 3H); ^13^C NMR (100 MHz, CDCl_3_) *δ* 203.4, 133.2, 123.1, 103.2, 83.5, 81.9, 79.4, 68.6, 47.5, 45.8, 45.4, 42.4, 28.2, 25.9, 24.7, 17.7, 13.6; IR (KBr) cm^-1^ 2953m, 2927m, 2869m, 2847m, 1736m, 1463w, 1178w, 1111s, 1076s, 1008m, 967s, 829m, 736m, 701m; HRMS (ESI^+^): C_17_H_23_BrO_4_ for [ M+Na]^+^, calculated 394.2608, found 393.06668; ${\left[\text{α}\right]}_{D}^{25}$ = + 2.450, (c 1.00, CHCl_3_).

### Removal of the chiral auxiliary on the cycloadduct

*p*-TsOH (17 mg, 0.089 mmol) was added to cycloadduct **3a** (26 mg, 0.089 mmol) in MeOH (1.8 mL) and the mixture was stirred at room temperature for 2h, Pyridine (11 µL, 0.13 mmol) was added and the mixture was concentrated under reduced pressure. The crude residue was purified using silica gel flash column chromatography [eluent: 20% EtOAc/Petroleum ether] to give compound **4** (11 mg, 88% yield) as a white solid. ^1^H NMR (400 MHz, CDCl_3_) *δ* 6.32 (t, *J* = 1.5 Hz, 2H), 5.10 (td, *J* = 3.6, 1.7 Hz, 2H), 4.39 (s, 1H), 2.91 (dd, *J* = 15.0, 4.9 Hz, 1H), 2.55 – 2.45 (m, 1H). ^13^C NMR (100 MHz, CDCl_3_) δ 206.5, 134.6, 131.8, 80.7, 78.9, 78.5, 45.1; HRMS (ESI^+^): C_7_H_8_O_3_ for [M+Na]^+^, calculated 163.0366, found 163.0367; ${[\text{α]}}_{\text{D}}^{20}$ = + 103.195, (c 0.10, CHCl_3_).

### Biological assays: the antiproliferative activity assay

#### Cell culture

T47D cells were maintained in RPMI 1640 supplemented with 10% FBS, and 100 units/ml penicillin-G and 100mg/ml streptomycin and 0.37% NaHCO_3_ and were incubated at 37°C in humidified air containing 5% CO_2_.

#### MTT method

T47D cells were planted into 96-well micro-plates at a density of 7×10^4^ cells/mL (100mL per well). After overnight incubation at 37°C, 100 µmol/L concentrations of test cycloadducts were added to the wells for preliminary screening and 5 different concentrations of test cycloadducts were added for GI_50_ testing. Cycloadducts were dissolved in DMSO before being diluted in the growth medium. The concentration of DMSO in the wells did not exceed 0.5%. Cells were further incubated for 96 h, at the end of the incubation time, fresh medium containing 0.5 mg/mL of MTT were added. Plates were incubated for another 4 h at 37 °C, the media was removed and formazan crystals formed in the cells were dissolved in 200mL of DMSO. Optical density was measured at 490 nm with DMSO as blank using an enzyme labeling instrument (Tecan, Switzerland). The inhibition ratio of viability for each concentration of the compounds was calculated with respect to the control and GI_50_ values were estimated with the software Graph pad Prism 7.04 Each experiment was repeated 3 times and the results were summarized in **Table 3** as mean ± SD (inmM).

**Table 3:** The antiproliferative effects of target compounds against T47D cells (*GI_50_*: μmol/L).

cycloadducts	*GI_50_* (μmol/L)
	T47D
**3a**	82.787
**3b**	94.555
**3c**	197.921
**3d**	267.736
**3e**	105.388
**3f**	136.534
**3g**	48.729
**3h**	87.803
**4**	79.701
Ribociclib	5.955

Ribociclib was used as positive control.

## Conclusion

A novel stereoselectivity allenyl ether (4 + 3) cycloaddition was explored for the construction of substituted cycloheptanones. The valuable approach was investigated by the camphor-derived chiral auxiliary inducing to substrates. Furthermore, the chiral auxiliary was removed *via p*-TsOH to generate corresponding enantiomer from the α-hydroxyl cycloheptanone in high yields. The bioactivities of these cycloadducts were evaluated, which had moderate inhibitory effect on breast cancer cell line T-47D.

## Data availability statement

The original contributions presented in the study are included in the article/**Supplementary Material**. Further inquiries can be directed to the corresponding authors.

## Author contributions

SH and HZ designed the experiment. YC, WL, HZ and YQ wrote the manuscript. WL, YS and AL performed experiments and analyzed the data. SH, YC and HZ supervised the entire project. All authors contributed to the article and approved the submitted version.
