# Supplementary material for: Stereoselective (4 + 3) cycloadditions of allenyl ethers-furans by chiral auxiliaries inducing and evaluation of anti-breast cancer activity
Source: Front Plant Sci. 2022 Dec 21;13:1087899. doi: 10.3389/fpls.2022.1087899 (PMC9812524; doi:10.3389/fpls.2022.1087899)
Supplement: Supplementary file 1 [file DataSheet_1.docx]

Stereoselective (4 + 3) Cycloadditions of Allenyl Ethers-Furans by Chiral Auxiliaries inducing and Evaluation of Anti-Breast Cancer Activity

Wenli Lei^1^, Yuyang Song^1^, Ai Long^1^, Yanyan Que^1^, Shuzhong He^1, 2^*, Hang Zhong^1^*, Yang Chen^3^*

1. School of Pharmaceutical Sciences, and Guizhou Engineering Laboratory for Synthetic Drugs, Guizhou University, Guiyang, Guizhou 550025, China.

2. State Key Laboratory of Functions and Applications of Medicinal Plants, Guizhou Medical University, Guiyang 550014, China.

3. State Key Laboratory Breeding Base of Green Pesticide and Agricultural Bioengineering, Key Laboratory of Green Pesticide and Agricultural Bioengineering, Ministry of Education, Guizhou University, Guiyang 550025, China

***Corresponding author**

Dr. Shuzhong He, E-mail: [szhe@gzu.edu.cn](mailto:szhe@gzu.edu.cn)

Dr. Hang Zhong, E-mail: kzhong@guz.edu.cn

Dr. Yang Chen, E-mail: [ychen1@gzu.edu.cn](mailto:ychen1@gzu.edu.cn)

**SUPPLEMENTARY INFORMATION**

**Supplementary Methods**

The detailed characterization data of intermediates and target compounds

**Supplementary Results**

Figures. S1. The ^1^H NMR and ^13^C NMR spectra of intermediates and target compounds

Table S1. X-ray crystallographic data of compound **3h** (CCDC 2215220).

**Supplementary Methods**

The detailed characterization data of intermediates and target compounds

**6** (1.2 g, 71% yield); ^1^H NMR (400 MHz, CDCl_3_) *δ* 4.57 – 4.39 (m, 1H), 4.11 (dd, *J* = 10.8, 1.2 Hz, 1H), 2.20 – 2.04 (m, 2H), 1.97 – 1.66 (m, 3H), 1.18 (s, 3H), 1.10 (s, 3H), 0.98 (s, 3H).

**Aux-6** (1.0 g, 83% yield); ^1^H NMR (400 MHz, CDCl_3_) *δ* 4.97 – 4.85 (m, 1H), 4.07 (dt, *J* = 10.9, 1.3 Hz, 1H), 3.52 (dd, *J* = 10.9, 2.5 Hz, 1H), 3.12 – 2.97 (m, 1H), 2.03 – 1.77 (m, 2H), 1.70 – 1.48 (m, 3H), 1.08 (s, 3H), 0.91 (s, 3H), 0.87 (s, 3H).

**7** (1.0 g, 81% yield); ^1^H NMR (400 MHz, CDCl_3_) *δ* 4.74 (d, *J* = 0.9 Hz, 1H), 4.33 (dd, *J* = 12.2, 2.4 Hz, 2H), 4.04 (d, *J* = 10.9 Hz, 1H), 3.53 (dd, *J* = 10.9, 2.5 Hz, 1H), 2.40 (d, *J* = 2.4 Hz, 1H), 2.07 (ddd, *J* = 13.2, 9.9, 4.6 Hz, 1H), 1.94 – 1.73 (m, 1H), 1.69 – 1.45 (m, 2H), 1.41 – 1.20 (m, 1H), 1.09 (s, 3H), 0.90 (s, 3H), 0.86 (s, 3H).

**1f** (0.9 g, 90% yield); ^1^H NMR (400 MHz, CDCl_3_) *δ* 6.70 (t, *J* = 6.0 Hz, 1H), 5.42 (dd, *J* = 12.9, 6.1 Hz, 2H), 4.82 (s, 1H), 4.06 (d, *J* = 10.9 Hz, 1H), 3.59 (dd, *J* = 10.9, 2.4 Hz, 1H), 2.17 (s, 1H), 1.96 – 1.78 (m, 1H), 1.69 – 1.52 (m, 3H), 1.19 – 0.76 (m, 9H).

**3a**: 26 mg (64%); yellow oil; ^1^H NMR (400 MHz, CDCl_3_) *δ* 6.31 (ddd, *J* = 26.9, 6.1, 1.7 Hz, 2H), 5.08 (dd, *J* = 5.2, 1.8 Hz, 1H), 5.00 (d, *J* = 5.0 Hz, 1H), 4.81 (s, 1H), 4.39 (d, *J* = 5.2 Hz, 1H), 3.99 (d, *J* = 10.9 Hz, 1H), 3.50 (dd, *J* = 10.9, 2.5 Hz, 1H), 2.75 (dd, *J* = 15.5, 4.9 Hz, 1H), 2.34 (d, *J* = 15.5 Hz, 1H), 2.06 (td, *J* = 9.8, 5.0 Hz, 1H), 1.91 – 1.76 (m,1H), 1.67 – 1.45 (m, 3H), 1.06 (s, 3H), 0.94 (s, 3H), 0.89 (s, 3H); ^13^C NMR (100 MHz, CDCl_3_) *δ* 204.5, 134.2, 132.4, 103.2, 82.5, 80.7, 78.2, 68.4, 47.4, 45.8, 45.4, 42.5, 28.5, 26.0, 24.7, 17.7, 13.6; IR (KBr) cm^-1^ 2962m, 2924m, 2869m, 1725m, 1463w, 1361w, 1322w, 1258w, 1175w, 1095s, 1012s, 964s, 804m, 723s; HRMS (ESI^+^): C_17_H_24_O_4_ for [M+Na]^+^, calculated 315.1572, found 315.1566; ${[\alpha]}_{D}^{25}$ = + 9.921 (c 1.00, CHCl_3_).

**3b**: 15 mg (52% yield); yellow oil; ^1^H NMR (400 MHz, CDCl_3_) *δ* 6.26 (dd, *J* = 5.9, 1.8 Hz, 1H), 6.07 (d, *J* = 5.9 Hz, 1H), 5.10 – 5.00 (m, 1H), 4.81 (s, 1H), 4.35 (d, *J* = 5.2 Hz, 1H), 3.98 (d, *J* = 10.9 Hz, 1H), 3.49 (dd, *J* = 10.9, 2.5 Hz, 1H), 2.55 (s, 1H), 2.39 (d, *J* = 15.3 Hz, 1H), 2.10 – 1.99 (m, 1H), 1.91 – 1.77 (m, 1H), 1.63 – 1.52 (m, 3H), 1.49 (s, 3H), 1.05 (s, 3H), 0.93 (s, 3H), 0.88 (s, 3H); ^13^C NMR (100 MHz, CDCl_3_) *δ* 204.97, 137.38, 132.21, 103.07, 84.80, 81.21, 80.96, 68.40, 51.73, 47.42, 45.44, 42.54, 28.46, 25.95, 24.75, 22.77, 17.68, 13.62; IR (KBr) cm^-1^ 2962m, 2927m, 2866m,1725m, 1460w, 1383w, 1325w, 1178w, 1115s, 1076s, 1006s, 830w, 736m, 727m; HRMS (ESI^+^): C_18_H_26_O_4_ for [M+Na]^+^, calculated 329.3918, found 329.17248; ${[\alpha]}_{D}^{25}$ = + 17.105, (c 0.67, CHCl_3_)

**3c:** 21 mg (48% yield); yellow oil; ^1^H NMR (400 MHz, CDCl_3_) *δ* 6.27 (d, J = 6.0 Hz, 1H), 6.07 (d, *J* = 5.8 Hz, 1H), 5.07 (d, *J* = 5.2 Hz, 1H), 4.82 (s, 1H), 4.37 (d, *J* = 5.2 Hz, 1H), 3.98 (d, *J* = 11.0 Hz, 1H), 3.49 (d, *J* = 11.0 Hz, 1H), 2.55 (d, *J* = 15.2 Hz, 1H), 2.36 (d, *J* = 15.3 Hz, 1H), 2.07 (ddt, *J* = 14.8, 9.4, 5.3 Hz, 2H), 1.87 – 1.76 (m, 2H), 1.65 – 1.46 (m, 3H), 1.06 (s, 3H), 0.98 (t, *J* = 7.5 Hz, 3H), 0.94 (s, 3H), 0.89 (s, 3H); ^13^C NMR (100 MHz, CDCl_3_) *δ* 205.2, 136.0, 132.5, 103.1, 88.1, 81.6, 80.9, 68.4, 50.0, 47.4, 45.4, 42.5, 28.9, 28.5, 26.0, 24.8, 17.7, 13.6, 8.8; IR (KBr) cm^-1^ 2959m, 2924m, 2850m, 1726m, 1470m, 1169w, 1120s, 1069s, 1008s, 967w, 826w, 804w, 733s; HRMS (ESI^+^): C_19_H_28_O_4_ for [M+Na]^+^, calculated 343.4188, found 343.18769; ${[\alpha]}_{D}^{25}$= + 16.221, (c 1.00, CHCl_3_).

**3d:** 22 mg (47% yield); yellow oil; ^1^H NMR (400 MHz, CDCl_3_) *δ* 6.26 (dd, *J* = 5.9, 1.8 Hz, 1H), 6.08 (d, *J* = 6.0 Hz, 1H), 5.06 (dd, *J* = 5.2, 1.9 Hz, 1H), 4.82 (s, 1H), 4.37 (d, *J* = 5.2 Hz, 1H), 3.99 (d, *J* = 10.9 Hz, 1H), 3.50 (dd, *J* = 10.9, 2.5 Hz, 1H), 2.54 (s, 1H), 2.38 (s, 1H), 2.11 – 2.03 (m, 2H), 1.87 – 1.80 (m, 1H), 1.77 – 1.70 (m, 2H), 1.62 – 1.55 (m, 2H), 1.51 (m, 2H), 1.06 (s, 3H), 0.97 (s, 3H), 0.94 (m, 3H), 0.90 (d, *J* = 2.4 Hz, 3H); ^13^C NMR (100 MHz, CDCl_3_) *δ* 205.3, 136.3, 132.2, 103.1, 87.7, 81.6, 80.8, 68.4, 50.3, 47.4, 45.4, 42.5, 38.3, 28.5, 26.0, 24.8, 17.7, 17.4, 14.4, 13.6; IR (KBr) cm^-1^ 2961w, 2930s, 2852w, 1730m, 1604w, 1513w, 1467m, 1393m, 1263m, 1183w, 1085s, 1008s, 962m, 825m, 795m, 764m, 706m, 661m, 559m; HRMS (ESI^+^): C_20_H_30_O_4_ for [M+Na]^+^, calculated 357.4458, found 357.20370; ${[\alpha]}_{D}^{20}$ = + 140.898, (c 0.20, CHCl_3_).

**3e:** 22 mg (43% yield); yellow oil; ^1^H NMR (400 MHz, CDCl_3_) *δ* 6.27 (dd, *J* = 6.0, 1.8 Hz, 1H), 6.08 (d, *J* = 6.0 Hz, 1H), 5.12 – 5.03 (m, 1H), 4.82 (s, 1H), 4.37 (d, *J* = 5.2 Hz, 1H), 3.99 (dd, *J* = 11.0, 1.3 Hz, 1H), 3.50 (dd, *J* = 10.9, 2.5 Hz, 1H), 2.56 (d, *J* = 15.2 Hz, 1H), 2.44 – 2.34 (m, 1H), 2.08 (ddd, *J* = 12.9, 9.8, 4.7 Hz, 2H), 1.90 – 1.78 (m, 1H), 1.74 – 1.48 (m, 3H), 1.06 (s, 3H), 0.95 (s, 4H), 0.93 – 0.85 (m, 8H); ^13^C NMR (100 MHz, CDCl_3_) *δ* 205.2, 136.4, 132.3, 103.1, 87.7, 81.6, 80.8, 68.4, 50.4, 47.4, 45.5, 42.5, 36.1, 32.1, 28.5, 26.0, 24.8, 23.7, 22.6, 17.7, 14.1, 13.6; IR (KBr) cm^-1^ 2957m, 2924m, 2877m, 1731m, 1492m, 1457m, 1396w, 1362w, 1309w,1274m, 1206w, 1184m, 1122m, 1082s, 972s, 893w, 817w, 738w, 708w, 601w; HRMS (ESI^+^): C_22_H_34_O_4_ for [M+Na]^+^, calculated 385.4998 found 385.47654; ${[\alpha]}_{D}^{20}$ = + 121.312, (c 0.10, CHCl_3_).

**3f:** 17 mg (35% yield); yellow oil; ^1^H NMR (400 MHz, CDCl_3_) *δ* 6.34 (dd, *J* = 6.0, 1.8 Hz, 1H), 6.14 (d, *J* = 6.0 Hz, 1H), 5.12 (d, *J* = 5.3 Hz, 1H), 4.82 (s, 1H), 4.39 (d, *J* = 5.2 Hz, 1H), 3.99 (d, *J* = 10.9 Hz, 1H), 3.66 (s, 2H), 3.61 (dd, *J* = 7.0, 4.4 Hz, 1H), 3.51 (d, *J* = 2.5 Hz, 1H), 2.77 (d, *J* = 15.5 Hz, 1H), 2.38 (d, *J* = 15.4 Hz, 1H), 2.07 (ddd, *J* = 13.9, 9.7, 4.7 Hz, 1H), 1.84 (d, *J* = 6.0 Hz, 1H), 1.65 – 1.49 (m, 3H), 1.06 (s, 3H), 0.94 (s, 3H), 0.92 – 0.85 (m, 9H); ^13^C NMR (100 MHz, CDCl_3_) *δ* 204.9, 134.7, 133.1, 103.2, 87.3, 81.4, 81.2, 72.3, 68.4, 67.4, 47.4, 47.3, 45.4, 42.5, 28.5, 25.9, 24.8, 17.7, 15.0, 13.6; IR (KBr) cm^-1^ 2963m, 2959m, 2857m, 1724m, 1467m, 1387w, 1263m, 1078s, 1011m, 965w,829m, 801m, 794m; HRMS (ESI^+^): C_20_H_30_O_5_ for [M+Na]^+^, calculated 373.4448, found 373.19847; ${[\alpha]}_{D}^{20}$= + 3.831, (c 0.10, CHCl_3_).

**3g:** 23 mg (45% yield); yellow oil; ^1^H NMR (400 MHz, CDCl_3_) *δ* 6.38 (d, *J* = 1.8 Hz, 1H), 6.20 (d, *J* = 6.0 Hz, 2H), 5.16 (dd, *J* = 5.2, 1.8 Hz, 2H), 5.05 (s, 2H), 4.82 (s, 2H), 4.39 (d, *J* = 5.2 Hz, 2H), 4.10 – 3.94 (m, 6H), 3.49 (dd, *J* = 11.0, 2.5 Hz, 2H), 2.76 (d, *J* = 15.5 Hz, 2H), 2.42 (d, *J* = 15.5 Hz, 2H), 2.12 – 2.00 (m, 3H), 1.83 (dt, *J* = 11.8, 5.8 Hz, 2H), 1.63 – 1.50 (m, 5H), 1.06 (s, 6H), 0.94 (s, 7H), 0.90 (d, *J* = 2.5 Hz, 11H); ^13^C NMR (100 MHz, CDCl_3_) *δ* 204.4, 133.7, 133.4, 103.2, 103.1, 88.2, 81.5, 81.3, 68.4, 65.9, 65.63, 5.40, 45.4, 44.8, 42.5, 28.5, 25.9, 24.7, 17.69, 13.6; IR (KBr) cm^-1^ 2953m, 2918m, 2871m, 2853m, 1726m, 1467w, 1373w, 1315w, 1257w, 1176w, 1109s, 1078s, 1014m, 964m, 938m, 838w, 798w, 740m, 613w, 491w; HRMS (ESI^+^): C_20_H_28_O_6_ for [M+Na]^+^, calculated 387.4278, found 387.17796; ${[\alpha]}_{D}^{20}$ = + 73.604, (c 0.12, CHCl_3_).

**3h:** 21 mg (40% yield); white solid; mp 80.7~81.2°C; ^1^H NMR (400 MHz, CDCl_3_) *δ* 6.38 (d, J = 2.0 Hz, 1H), 4.97 (d, J = 5.1 Hz, 1H), 4.95 – 4.93 (m, 1H), 4.84 (s, 1H), 4.52 (d, *J* = 5.1 Hz, 1H), 4.00 (d, *J* = 10.9 Hz, 1H), 3.52 (dd, *J* = 10.9, 2.5 Hz, 1H), 2.77 (d, *J* = 4.8 Hz, 1H), 2.43 (d, *J* = 15.7 Hz, 1H), 2.24 (ddd, *J* = 13.8, 9.9, 4.6 Hz, 1H), 1.97 – 1.78 (m, 1H), 1.69 – 1.42 (m, 3H), 1.06 (s, 3H), 0.97 (s, 3H), 0.90 (s, 3H); ^13^C NMR (100 MHz, CDCl_3_) *δ* 203.4, 133.2, 123.1, 103.2, 83.5, 81.9, 79.4, 68.6, 47.5, 45.8, 45.4, 42.4, 28.2, 25.9, 24.7, 17.7, 13.6; IR (KBr) cm^-1^ 2953m, 2927m, 2869m, 2847m, 1736m, 1463w, 1178w, 1111s, 1076s, 1008m, 967s, 829m, 736m, 701m; HRMS (ESI^+^): C_17_H_23_BrO_4_ for [M+Na]^+^, calculated 394.2608, found 393.06668; ${[\alpha]}_{D}^{25}$ = + 2.450, (c 1.00, CHCl_3_).

**4** (11 mg, 88% yield) as a white solid. ^1^H NMR (400 MHz, CDCl_3_) δ 6.32 (t, *J* = 1.5 Hz, 2H), 5.10 (td, *J* = 3.6, 1.7 Hz, 2H), 4.39 (s, 1H), 2.91 (dd, *J* = 15.0, 4.9 Hz, 1H), 2.55 – 2.45 (m, 1H); ${[\alpha]}_{D}^{20}$= + 103.195, (c 0.10, CHCl_3_).

**Supplementary Results**

Figures. S1. The ^1^H NMR and ^13^C NMR spectra of intermediates and target compounds

Table S1. X-ray crystallographic data of compound **3h** (CCDC 2215220).


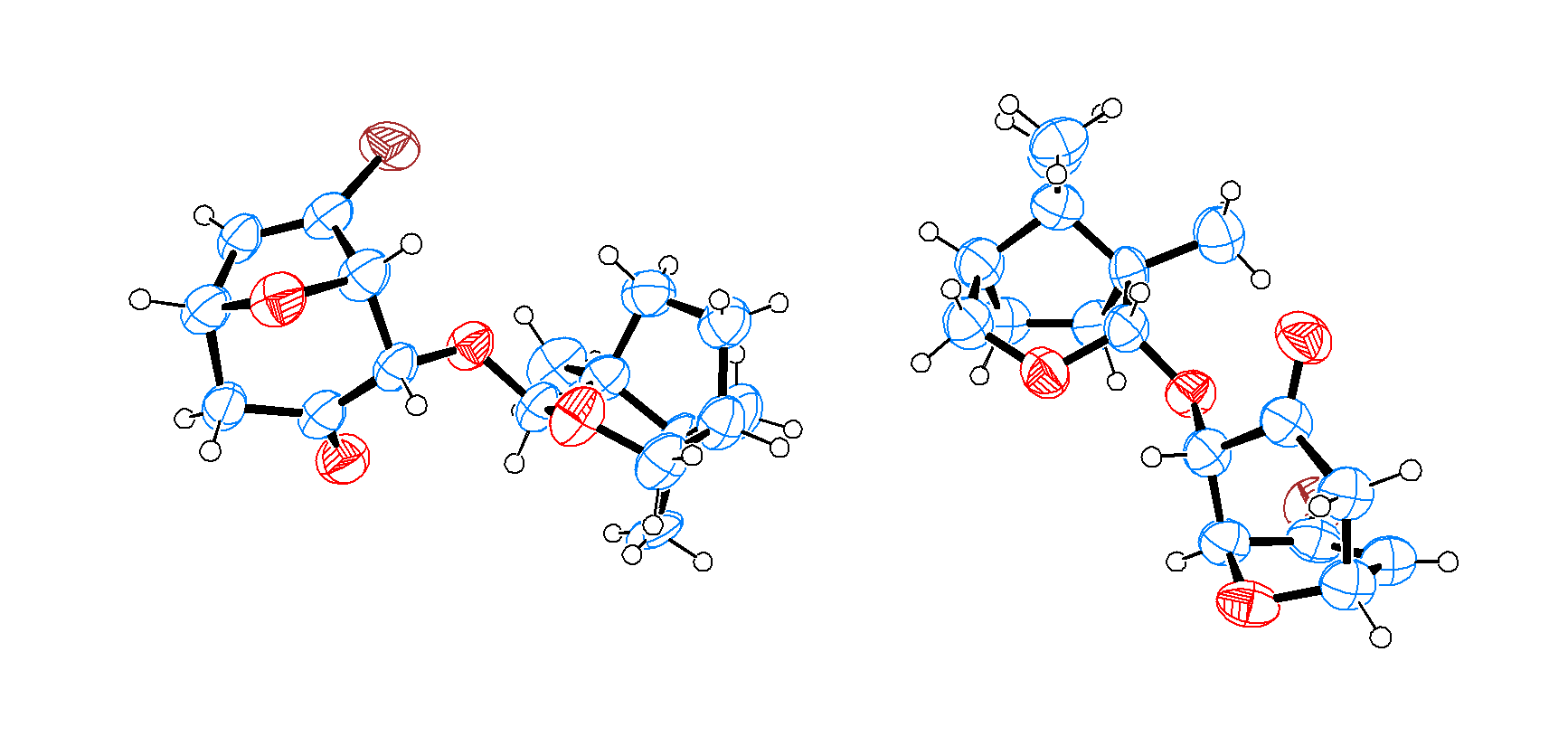


| Identification code | 221013a |
| --- | --- |
| Empirical formula | C_17_H_23_BrO_4_ |
| Formula weight | 371.26 |
| Temperature/K | 298(2) K |
| Crystal system | Monoclinic |
| Space group | P2 (1) |
| Unit cell dimensions | a = 11.1920 (12) Å |
|  | b = 7.1811 (8) Å |
|  | c = 21.758 (2) Å |
| α/°, β/°, γ/° | 90°, 104.705(4) °, 90° |
| Volume | 1691.4(3) Å^3^ |
| Z | 4 |
| Density (calculated) | 1.458 mg / m^-3^ |
| μ / mm^‑1^ | 2.445 |
| F (000) | 768 |
| Crystal size | 0.27 × 0.07 × 0.04 mm^3^ |
| 2Θ range for data collection | 1.94° to 28.44° |
| Index ranges | −14 ≤ h ≤ 14, −9 ≤ k ≤ 9, −28 ≤ l ≤ 29 |
| Reflections collected | 7217 |
| Independent reflections | 7230[ R (int) = 0.0000] |
| Data / restraints / parameters | 7230 / 1379 / 406 |
| Goodness-of-fit on F^2^ | 1.084 |
| Final R indexes [I>2σ (I) i.e.F_0_>4σ (F_0_)] | R_1_ = 0.1030, wR_2_ = 0.2845 |
| Final R indexes [all data] | R_1_ = 0.2062, wR_2_ = 0.3382 |
| Absolute structure parameter | 0.01(3) |
| Extinction coefficient | 0.010(3) |
| Largest diff. peak/hole/ e Å^-3^ | 0.676/-0.697 |
